# Tracheobronchitis Due to Invasive Aspergillus fumigatus in a Male With HIV With Minimal Imaging Findings

**DOI:** 10.7759/cureus.18806

**Published:** 2021-10-15

**Authors:** Calvin A He, Rory A Smith, Sharon Wang, Sarkis Arabian, Mufadda Hasan

**Affiliations:** 1 Pulmonary and Critical Care Medicine, Arrowhead Regional Medical Center, Colton, USA; 2 Infectious Disease, Arrowhead Regional Medical Center, Colton, USA

**Keywords:** invasive aspergillosis, fumigatus, aspergillus, tracheobronchitis, hiv

## Abstract

*Aspergillus *tracheobronchitis (AT) is a rare manifestation of invasive aspergillosis. We present a case of tracheobronchitis caused by *Aspergillus* *fumigatus* in a 33-year-old male with neutropenia and known human immunodeficiency virus (HIV) infection with acquired immunodeficiency syndrome (AIDS).

A 33-year-old male with HIV/AIDS presented to the hospital with symptoms of productive cough for over two months associated with subjective fevers, chills, and body aches. Computed tomography (CT) of the chest was significant for scattered sub-centimeter bilateral upper lobe nodules but otherwise normal. The patient underwent an extensive evaluation for his respiratory symptoms, including an initial sputum culture, which grew *Haemophilus* *parainfluenzae* in addition to preliminary fungal growth, prompting further evaluation with bronchoscopy. Bronchoscopy revealed diffuse adherent obstructive pseudomembranous plaques in the trachea and bilateral upper lobe segmental bronchi. Bronchoalveolar lavage cultures later grew *Aspergillus fumigatus*. Tracheobronchitis due to *Aspergillus* species is a rare cause of infection in patients with HIV. We believe that this case underscores the importance of further evaluation utilizing bronchoscopy in patients with AIDS who have respiratory symptoms despite mild abnormalities on chest CT. This approach can be used to rule out atypical endobronchial infections such as tracheobronchitis due to *Aspergillus* species.

## Introduction

Invasive *Aspergillus *is a major cause of morbidity and mortality in the immunocompromised population [1]. Tracheobronchitis due to *Aspergillus *infection is relatively uncommon, occurring in less than 7% of pulmonary aspergillosis cases [2]. *Aspergillus *infection is rare in the population of patients with human immunodeficiency virus (HIV); however, when present, it is frequently associated with neutropenia [3]. Respiratory complaints in a patient with HIV and acquired immunodeficiency syndrome (AIDS) can represent a multitude of infectious agents, and tracheobronchitis due to *Aspergillus *species is often not considered as part of the initial differential diagnosis. We report a case of *Aspergillus *tracheobronchitis (AT) in a 33-year-old male with HIV/AIDS and neutropenia diagnosed by bronchoscopy.

## Case presentation

A 33-year-old male with HIV diagnosed four years prior presented to the hospital with complaints of cough productive of white sputum, fever, and body aches for the past two months. The patient was seen at an outside hospital two months prior and diagnosed with pneumonia. The causative agent of the pneumonia was not identified, and the patient denied taking antifungal therapy. He reported undergoing a bronchoscopy during the previous hospitalization but was unsure of the results. The patient was discharged with antibiotics; however, the cough and fever did not improve.

Approximately a year ago, the patient commenced the regimen elvitegravir/cobicistat/emtricitabine/tenofovir alafenamide for HIV infection and sulfamethoxazole/trimethoprim for *Pneumocystis jiroveci* pneumonia (PJP) prophylaxis, endorsing compliance with these medications. He denied prior intravenous drug use and sexual intercourse with men. He was unsure of his last CD4+ lymphocyte count, stating it was “low.” Serology also found no evidence of hepatitis B or C virus coinfection. The patient originally immigrated from Honduras approximately six years ago and worked as a barber.

**Figure 1:**
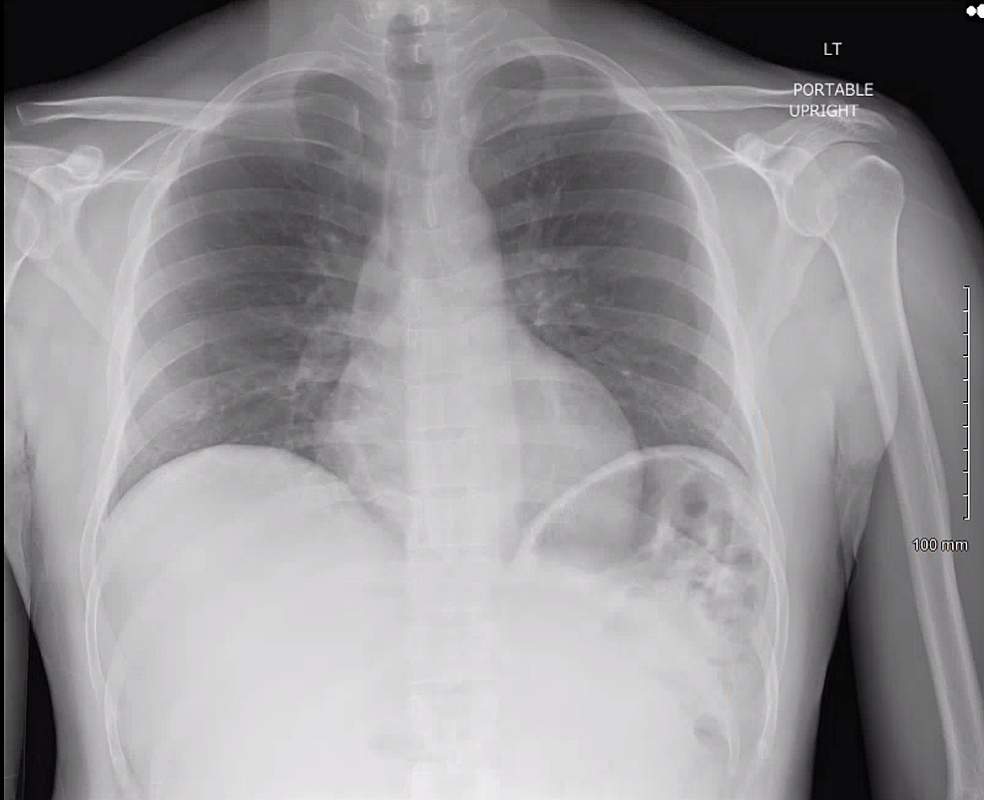
Unremarkable appearing upright chest radiograph.

**Figure 2:**
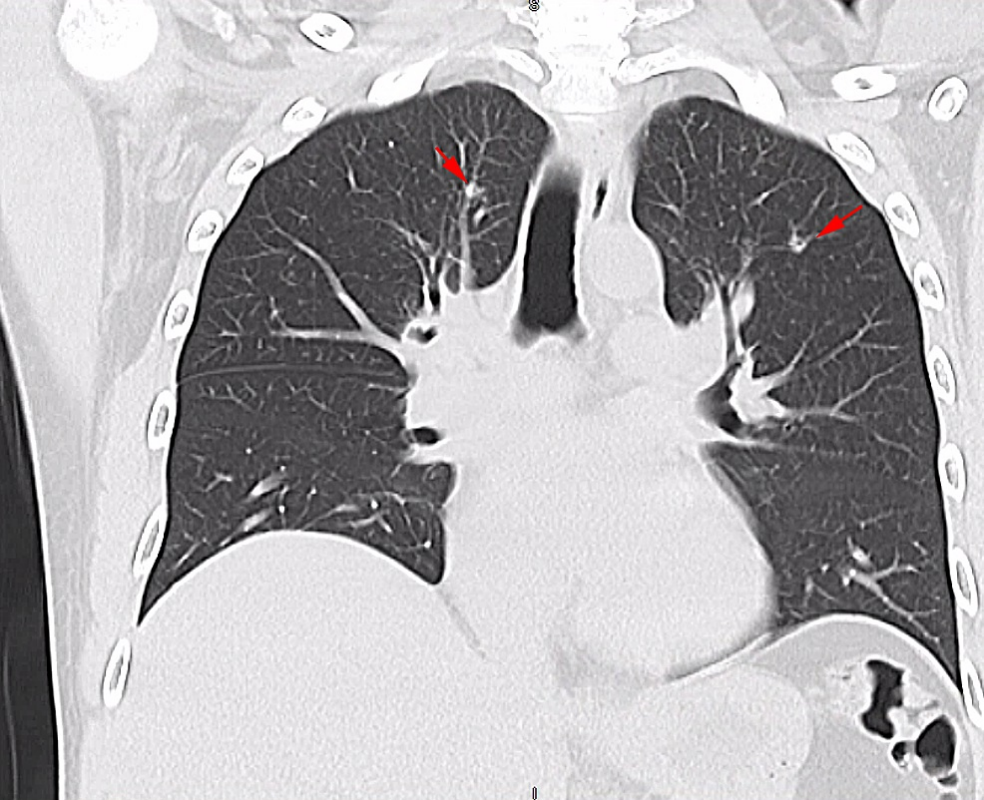
Computed tomography of the chest without contrast showing subtle scattered nodules in the bilateral upper lobes (arrows), more prominent in the left upper lobe.

On admission to this hospital, the patient was afebrile, tachycardic with a heart rate of 107 beats/minute, and saturating 98% on room air. He appeared nontoxic, and his respiratory examination was clear to auscultation bilaterally. Complete blood count consisted of a white blood cell count of 3900/mm^3^, bands of 2%, hemoglobin of 11.2 g/dL, and platelets of 768,000/mm^3^. The absolute neutrophil count was 234 cells/uL, indicating severe neutropenia. A basic metabolic panel was normal. The chest radiograph was unremarkable (Figure 1). A computed tomography (CT) of the chest without contrast revealed subtle scattered nodules in the bilateral upper lobes, more prominent in the left upper lobe (Figure 2).

Sputum cultures, including acid-fast bacilli (AFB) stains, and blood cultures were obtained. The patient was started on empiric antibiotics with ceftriaxone and azithromycin for seven and five days, respectively. He developed occasional febrile episodes, which persisted during the hospitalization in addition to productive cough despite completing the course of antibiotics. Significant eosinophilia developed with a peak absolute eosinophil count of 783 cells/uL. His CD4+ lymphocyte count returned at 33 cells/uL, and his HIV viral load was found to be 49,700 copies/mL.

Neutropenia persisted, and a hematologist recommended replacing sulfamethoxazole/trimethoprim with atovaquone for PJP prophylaxis, repleting B12 levels that were slightly low at 245 pg/mL, and obtaining a bone marrow biopsy, which was nondiagnostic. Three AFB sputum smears were negative. *Coccidioides *antibody complement fixation was negative at <1:2. Notably, *Aspergillus fumigatus* antibodies and beta-d-glucan were not detected in serum. Sputum culture resulted in moderate *Haemophilus parainfluenzae* growth and scant preliminary fungal growth. Outside records were obtained and revealed that the patient had undergone bronchial washings growing *Aspergillus fumigatus*. Unfortunately, the operative report was unavailable. Due to the patient’s symptoms being inconsistent with the minimal imaging findings, a repeat bronchoscopy was performed at our facility.

**Figure 3:**
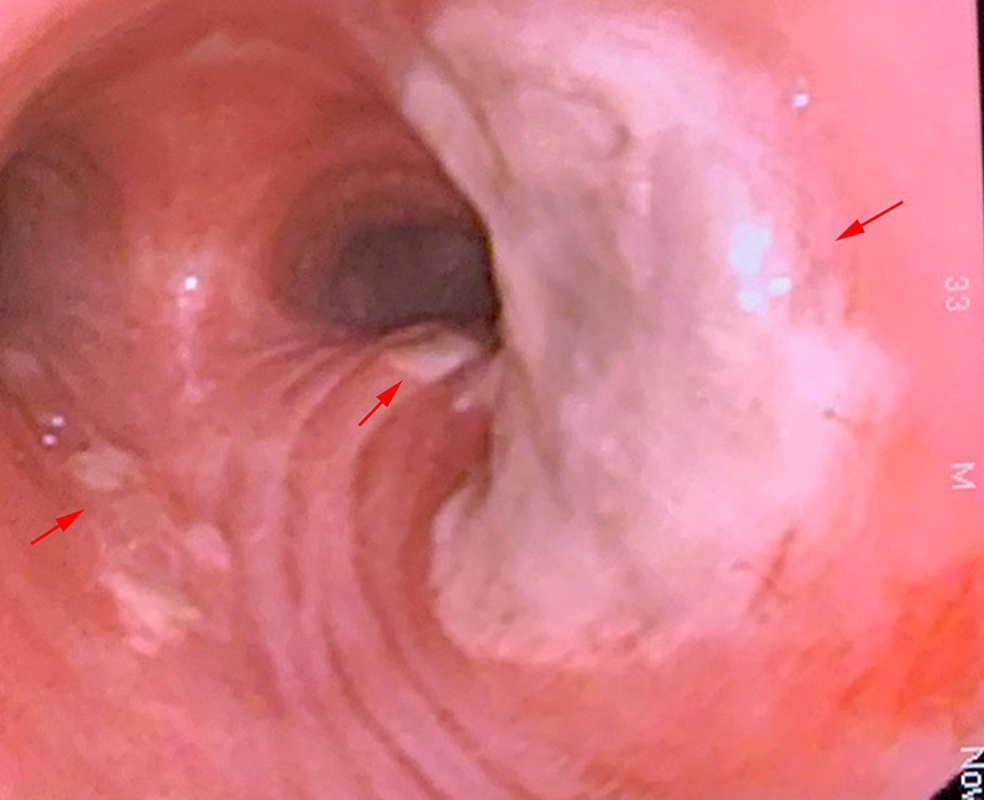
Thick white mucopurulent secretions in the distal trachea seen via bronchoscopy (arrows).

**Figure 4:**
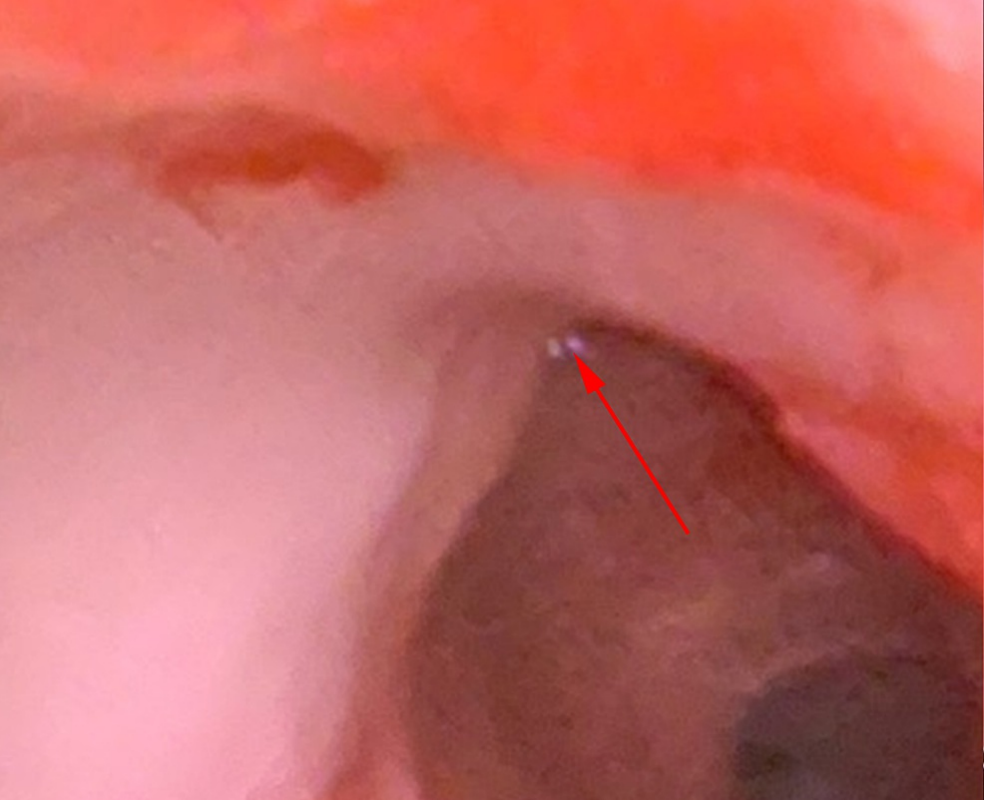
Bronchoscopy showing that the left superior lobar bronchus was completely obstructed with white plaques.

**Figure 5:**
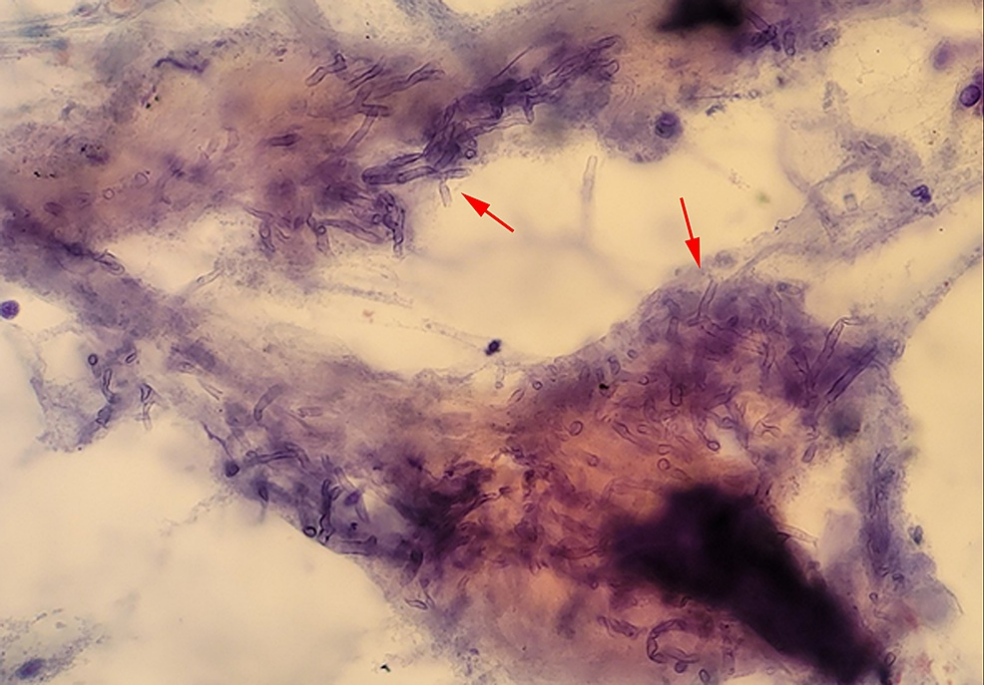
Bronchial brushings with Papanicolaou’s stain revealing branching hyphae consistent with Aspergillus (red arrow).

During bronchoscopy, immediately after intubation of the trachea, thick whitish-brown adherent plaques were noted in the distal trachea (Figure 3). Additionally, the right upper lobe segmental bronchi had thick adherent white plaques, and the left superior lobar bronchus was completely obstructed with white plaques (Figure 4). Multiple forceps biopsies were taken from the plaques in addition to brushings. Bronchoalveolar lavage (BAL) was performed in the right upper and left upper lobe segments. BAL cell count was found to have 90% neutrophils, 7% macrophages, and 3% lymphocytes. BAL studies showed positive galactomannan with an index value of 7.7, and a later culture grew *Aspergillus fumigatus*. Pathology resulted in tracheobronchial hyaline cartilage with mucosa thickly encrusted with matted *Aspergillus *species (Figure 5).

The patient was started on voriconazole 200 mg (4 mg/kg) orally twice a day with plans for a prolonged treatment course of six to 12 months for invasive aspergillosis. Antiretroviral therapy was replaced with dolutegravir and emtricitabine/tenofovir alafenamide due to the drug-drug interaction between voriconazole and cobicistat. Sulfamethoxazole/trimethoprim was replaced with atovaquone for PJP prophylaxis due to concerns of neutropenia caused by bone marrow suppression. The patient’s fever and respiratory status improved with the initiation of antifungal medication. The patient was discharged home with close outpatient follow-up. At two months, he followed up with the pulmonary clinic and reported significant improvement in his respiratory symptoms as well as continued compliance with antifungal and HIV medications. Unfortunately, no laboratory data was available at the time of the follow-up.

## Discussion

Respiratory symptoms with minimal chest imaging findings in a patient with AIDS pose a unique clinical dilemma as the differential diagnosis in such an immunocompromised patient is exceedingly broad. The differential diagnosis for pneumonia in a patient with AIDS includes bacterial, mycobacterial, fungal, viral, and parasitic infections [4]. Bacterial pneumonias are currently the most common cause of pneumonia in this population, and *Streptococcus pneumoniae* is the most frequent organism implicated [5]. Aspergillosis as a cause of pneumonia in patients with AIDS is rarely at the top of the differential diagnosis. Tracheobronchitis due to *Aspergillus *was not entertained as a diagnosis for our patient until sputum cultures returned with fungal growth coupled with a lack of improvement with empiric antibiotics.

Isolated AT is an uncommon presentation of invasive aspergillosis [6]. Aspergillosis infection is rare in patients with HIV; however, when present, it is commonly associated with neutropenia and a CD4 count of <100 cells/mm^3^, both present in this patient [3,6]. In reviewing AT cases, the most frequently associated conditions were solid organ transplantation (44.2%), hematologic malignancy (21.2%), neutropenia (18.7%), and chronic obstructive pulmonary disease (15.4%) [7]. Another case series reported malignancy as the most common underlying comorbidity in patients with AT [6]. Nonspecific respiratory symptoms such as productive cough, fever, and dyspnea are the most common symptoms associated with AT and were all noted in our patient [7]. CT imaging for AT may show tracheobronchial wall thickening, atelectasis, or an endobronchial mass, although CT is not always useful. Chest CT was reported to have specific findings for AT in only 25% of cases [7]. The nonspecific clinical presentation and nonspecific imaging findings can potentially lead to a delay in the diagnosis of AT [7].

Bronchoscopy has a high diagnostic yield for AT since sputum cultures lack sensitivity and specificity for invasive aspergillosis in the immunocompromised population [7]. In a case series of four patients with AIDS with AT, all four were diagnosed with bronchoscopy [2]. The threshold to perform diagnostic bronchoscopy should be low when suspecting invasive aspergillosis in an immunocompromised patient with minimal abnormalities on chest imaging. Several forms of AT have been described, including obstructive bronchial aspergillosis, ulcerative tracheobronchitis, and pseudomembranous tracheobronchitis [5]. Our patient had features of both obstructive and pseudomembranous tracheobronchitis.

This case raises multiple relevant clinical points. Our patient with HIV/AIDS had severe neutropenia possibly predisposing him to infection with *Aspergillus*. Neutropenia is a known risk factor for invasive aspergillosis [8]. In a prior case series, three of four patients with AIDS with *Aspergillus *tracheobronchitis were neutropenic [2]. According to a review of invasive aspergillosis, the most important risk factor for invasive aspergillosis in patients with AIDS was neutropenia, often secondary to medications such as sulfamethoxazole/trimethoprim [9]. Moreover, HIV infection is associated with a profound effect on hematopoiesis with anemia, thrombocytopenia, and neutropenia, all well described in infected patients [10]. The exact etiology of neutropenia in our patient was never discovered. Given the patient’s high viral load, we theorize that the potential etiology was likely due to prior poor compliance with HIV medications coupled with sulfamethoxazole/trimethoprim. It is probable that our patient’s severe neutropenia contributed to infection with AT. Additionally, the patient developed marked peripheral eosinophilia, an association not only established with other forms of *Aspergillus *disease, such as allergic bronchopulmonary aspergillosis, but also described in invasive aspergillosis infections [11].

In terms of treatment for AT, there are no specific recommendations; however, the first-line agent for invasive aspergillosis is voriconazole [7]. Our patient was placed on voriconazole and demonstrated clinical improvement both during the hospitalization and two months post-diagnosis.

## Conclusions

In conclusion, respiratory symptoms in an immunocompromised patient represent a unique challenge for the clinician, particularly when chest imaging is nonspecific. AT is a rare manifestation of invasive aspergillosis and mandates bronchoscopic evaluation for definitive diagnosis. This case of AT in a patient with AIDS and neutropenia highlights the importance of considering bronchoscopy early to evaluate for endobronchial infections, particularly when chest imaging is not indicative of a specific diagnosis in a severely immunocompromised patient.

## References

[1] Meersseman W, Lagrou K, Maertens J, Van Wijngaerden E (2007). Invasive aspergillosis in the intensive care unit. Clin Infect Dis.

[2] Kemper CA, Hostetler JS, Follansbee SE (1993). Ulcerative and plaque-like tracheobronchitis due to infection with Aspergillus in patients with AIDS. Clin Infect Dis.

[3] Holding KJ, Dworkin MS, Wan PC, Hanson DL, Klevens RM, Jones JL, Sullivan PS (2000). Aspergillosis among people infected with human immunodeficiency virus: incidence and survival. Adult and Adolescent Spectrum of HIV Disease Project. Clin Infect Dis.

[4] Huang L, Crothers K (2009). HIV-associated opportunistic pneumonias. Respirology.

[5] Benito N, Moreno A, Miro JM, Torres A (2012). Pulmonary infections in HIV-infected patients: an update in the 21st century. Eur Respir J.

[6] Wu N, Huang Y, Li Q, Bai C, Huang HD, Yao XP (2010). Isolated invasive Aspergillus tracheobronchitis: a clinical study of 19 cases. Clin Microbiol Infect.

[7] Fernández-Ruiz M, Silva JT, San-Juan R (2012). Aspergillus tracheobronchitis: report of 8 cases and review of the literature. Medicine (Baltimore).

[8] Kosmidis C, Denning DW (2015). The clinical spectrum of pulmonary aspergillosis. Thorax.

[9] Baddley JW (2011). Clinical risk factors for invasive aspergillosis. Med Mycol.

[10] Levine AM, Karim R, Mack W (2006). Neutropenia in human immunodeficiency virus infection: data from the women’s interagency HIV study. Arch Intern Med.

[11] Parta M, Dimitrova D, Freeman AF (2019). Invasive and allergic complications due to Aspergillus fumigatus in allogenic hematopoietic cell transplantation (HCT) primary immunodeficiency (PID) patients. Biol Blood Marrow Tr.

